# aTAP: automated transcriptome analysis platform for processing RNA-seq data by *de novo* assembly

**DOI:** 10.1016/j.heliyon.2022.e10255

**Published:** 2022-08-15

**Authors:** Komwit Surachat, Todd Duane Taylor, Wanicbut Wattanamatiphot, Sukgamon Sukpisit, Kongpop Jeenkeawpiam

**Affiliations:** a Department of Biomedical Sciences and Biomedical Engineering, Faculty of Medicine, Prince of Songkla University, Hat Yai, Songkhla 90110, Thailand; b Translational Medicine Research Center, Faculty of Medicine, Prince of Songkla University, Hat Yai, Songkhla 90110, Thailand; c Molecular Evolution and Computational Biology Research Unit, Faculty of Science, Prince of Songkla University, Hat Yai, Songkhla 90110, Thailand; d RIKEN Center for Integrative Medical Sciences, Yokohama, Kanagawa 230-0045, Japan; e Division of Computational Science, Faculty of Science, Prince of Songkla University, Hat Yai, Songkhla 90110, Thailand

**Keywords:** Transcriptome, RNA-seq, Bioinformatics workflow, Differentially expressed genes, Gene expression profile

## Abstract

RNA-seq is a sequencing technique that uses next-generation sequencing (NGS) to explore and study the entire transcriptome of a biological sample. NGS-based analyses are mostly performed via command-line interfaces, which is an obstacle for molecular biologists and researchers. Therefore, the higher throughputs from NGS can only be accessed with the help of bioinformatics and computer science expertise. As the cost of sequencing is continuously falling, the use of RNA-seq seems certain to increase. To minimize the problems encountered by biologists and researchers in RNA-seq data analysis, we propose an automated platform with a web application that integrates various bioinformatics pipelines. The platform is intended to enable academic users to more easily analyze transcriptome datasets. Our automated Transcriptome Analysis Platform (aTAP) offers comprehensive bioinformatics workflows, including quality control of raw reads, trimming of low-quality reads, de novo transcriptome assembly, transcript expression quantification, differential expression analysis, and transcript annotation. aTAP has a user-friendly graphical interface, allowing researchers to interact with and visualize results in the web browser. This project offers an alternative way to analyze transcriptome data, by integrating efficient and well-known tools, that is simpler and more accessible to research communities. aTAP is freely available to academic users at https://atap.psu.ac.th/.

## 1 Introduction

RNA sequencing (RNA-seq) is a technique used to investigate the RNA sequences and quantify gene expression levels in a biological sample using next-generation sequencing (NGS) technology [1, 2]. It is widely used to study cellular transcriptomes under specific conditions and at particular times in target organisms [3]. RNA-seq has been used to investigate gene expression profiles and biological processes in the fields of medicine [4, 5], biotechnology [6, 7], and molecular ecology [8].

With the rapid development of NGS and third-generation sequencing technology, the quality and accuracy of base calls have been improved, data throughput has been increased, and cost per base has been reduced. Consequently, many researchers all around the world have proposed and conducted RNA-seq studies to prove their hypotheses and share new findings with their communities. However, the complexity of the data analysis can make it difficult for molecular biologists, laboratory scientists, and medical doctors to extract the relevant information from the vast data generated. Several bioinformatics tools have been created on open-source platforms and bundled with programming scripts with command-line interfaces (CLIs). Performing analyses with these tools requires computer programming and bioinformatics skills, from the data quality control to the visualization of the results. This can take time to learn, and when setting parameters, it is easy to make mistakes that are difficult to trace back. Therefore, a user-friendly interface for analyzing data is warranted.

Many bioinformatics pipelines have been proposed to help scientists extract gene expression profiles from experimental RNA-seq data but most of the pipelines are implemented to analyse only reference-based transcriptome data [9, 10, 11, 12]. This approach can be a problem in the absence of an annotated draft or complete genome. In addition, some pipelines have been designed and implemented in CLI form [12, 13, 14], making it difficult for novice users to execute the necessary commands and retrieve the results. In response, several tools have been proposed that allow the user to interact directly with a graphical user interface (GUI) instead of using the text-based commands. For example, TCC-GUI [15], GENAVi [16], and RNASeqGUI [17] are GUI-based bioinformatics tools that can perform normalization and differential expression analysis of transcriptome data. However, these tools either lack some important analyses for transcriptome assembly or still need to be installed with a Linux-based command line that could be difficult for non-bioinformaticians.

In this paper, we introduce an automated transcriptome analysis platform (aTAP) for analyzing RNA-seq data with a de novo assembly-based method using Trinity [18], Trinotate [19], and several open-source bioinformatics toolkits. aTAP is a web-based platform with a user-friendly GUI to minimize the programming and bioinformatics skills required for analysis. This tool can automatically process data in a single step and provide interactive and comprehensive reports and visualizations. In addition, experienced users can customize aTAP by configuring advanced parameters in each tool of the pipeline according to the user's requirements. aTAP also offers a ready-to-use setting for novice users. The results are presented in several interactive forms including tables, graphs, and word clouds, allowing users to explore and identify differential gene expression (DGE) at the gene or transcript level for each pairwise condition of an experiment. Additionally, the visualization of the results can be directly configured and exported into files and images. aTAP is proposed to simplify RNA-seq data analysis and make the software configuration less complicated. This web-based platform is freely available for academic users at https://atap.psu.ac.th/.

## 2 Materials and methods

### 2.1 aTAP implementation

aTAP is a web application implementing an automated analysis pipeline for analyzing RNA-seq data. The application integrates several open-source tools together with in-house developed scripts. aTAP is hosted by an Apache web server maintained by Prince of Songkla University, Thailand. We used PHP and MySQL relational databases to construct backend architecture that was installed in a computer cluster comprising three nodes. The Job Management System (JMS) [20] was installed to manage workflow and organize computer nodes within the cluster together with in-house scripts. Sixteen CPUs will be preserved for running each job and aTAP can run ten jobs at the same time on one computer node. The job queue is managed by PHP script for submission to the backend script to find a computer node and run the analysis pipeline. However, the results will be stored on the server only 30 days and then they will be automatically removed.

### 2.2 Bioinformatics pipeline

The aTAP system was built from various bioinformatics pipelines wrapped by a vast array of programing languages including shell script, python, R, JavaScript, and HTML. Public bioinformatics tools were integrated into aTAP to enable users to perform analysis from the beginning to the visualization process. In the first step of the pipeline, the quality of each sequence is evaluated by FastQC, and the report is then generated by MultiQC [21]. MultiQC can organize multiple QC reports from multiple files in the same window and let the user see an overview of the quality of all the sequences easily.

Before the de novo assembly process, the option to trim adapter and low-quality reads is available using Trimmomatic [22]. The de novo transcriptome assembly is then performed using Trinity [18]. The assembly quality is then assessed to identify the breadth of the genetic composition and transcript contiguity using blast+ and bowtie2 [23, 24, 25].

Furthermore, in the transcript quantification step, the user can select either alignment-based or alignment-free abundance estimation methods including RSEM [26], salmon [27], and Kallisto [28]. Differentially expressed genes and count normalization analysis are performed by DESeq2 [29] or edgeR [30] based on the user's sample. For non-replication experiments, only edgeR can be selected for the analysis, since edgeR supports having no biological replicates in the study with a fixed dispersion setting.

**Figure 1:**
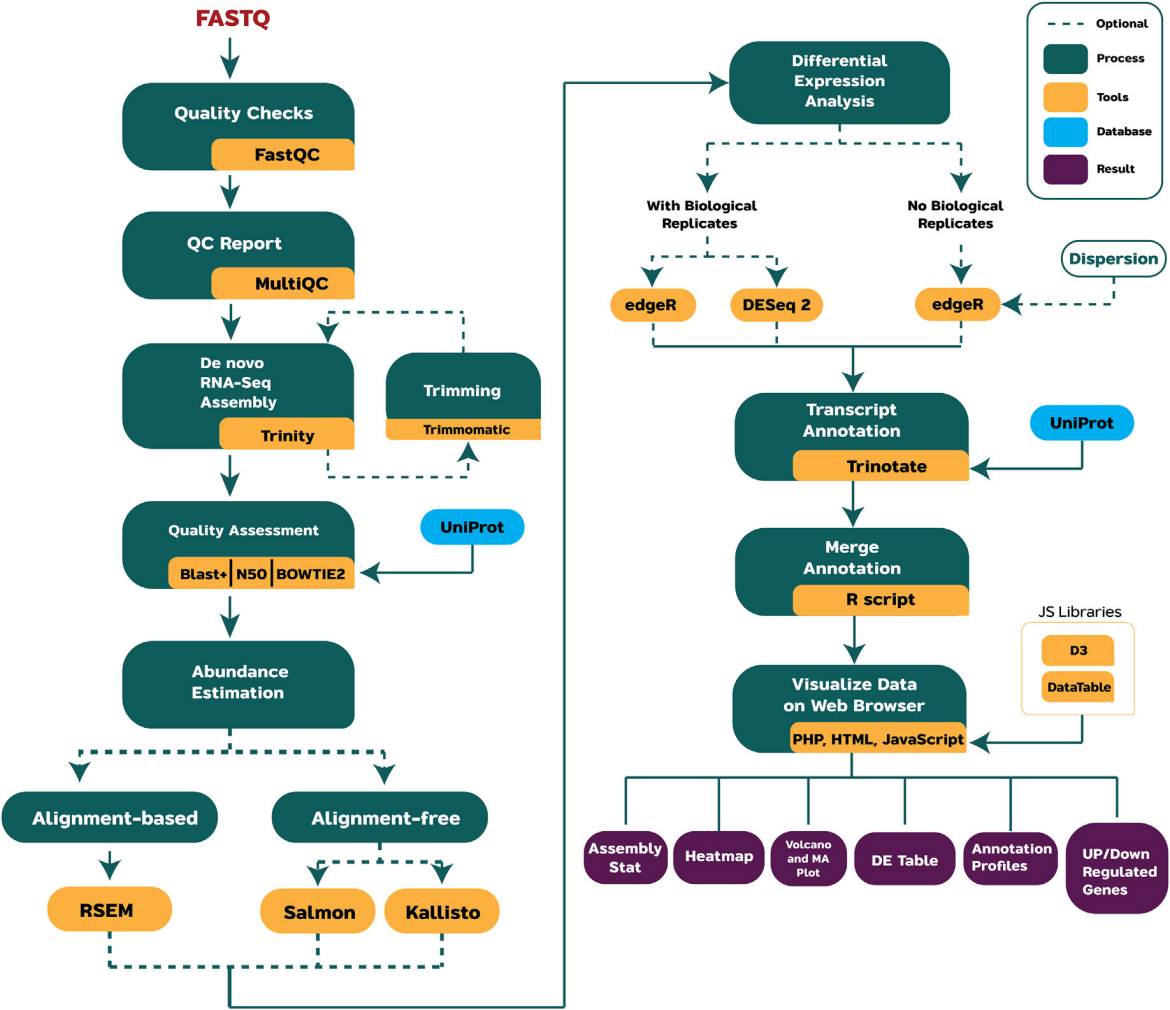
Bioinformatics workflow of analysis steps performed by the aTAP pipeline.

Finally, the assembled transcripts are annotated through several steps to identify coding regions within transcripts and determine homology search, protein family, and domain profiles using TransDecoder (https://github.com/TransDecoder/TransDecoder), BLAST+ [31], HMMER [32, 33], and PFAM [34, 35], respectively. These annotation steps were wrapped up into a single pipeline with Trinotate [19]. The annotation profiles are based on the UniProt database [36]. The overall bioinformatics steps are shown in Figure 1.

### 2.3 Web interface and system architecture

The web interface of aTAP was created using the front-end open-source toolkit, Bootstrap framework (https://getbootstrap.com/). This toolkit is integrated with several computer languages, including PHP: Hypertext Preprocessor (PHP), HyperText Markup Language 5, CSS3 standards, and, to wrap up the interactive graphical user interface, JavaScript.

**Figure 2:**
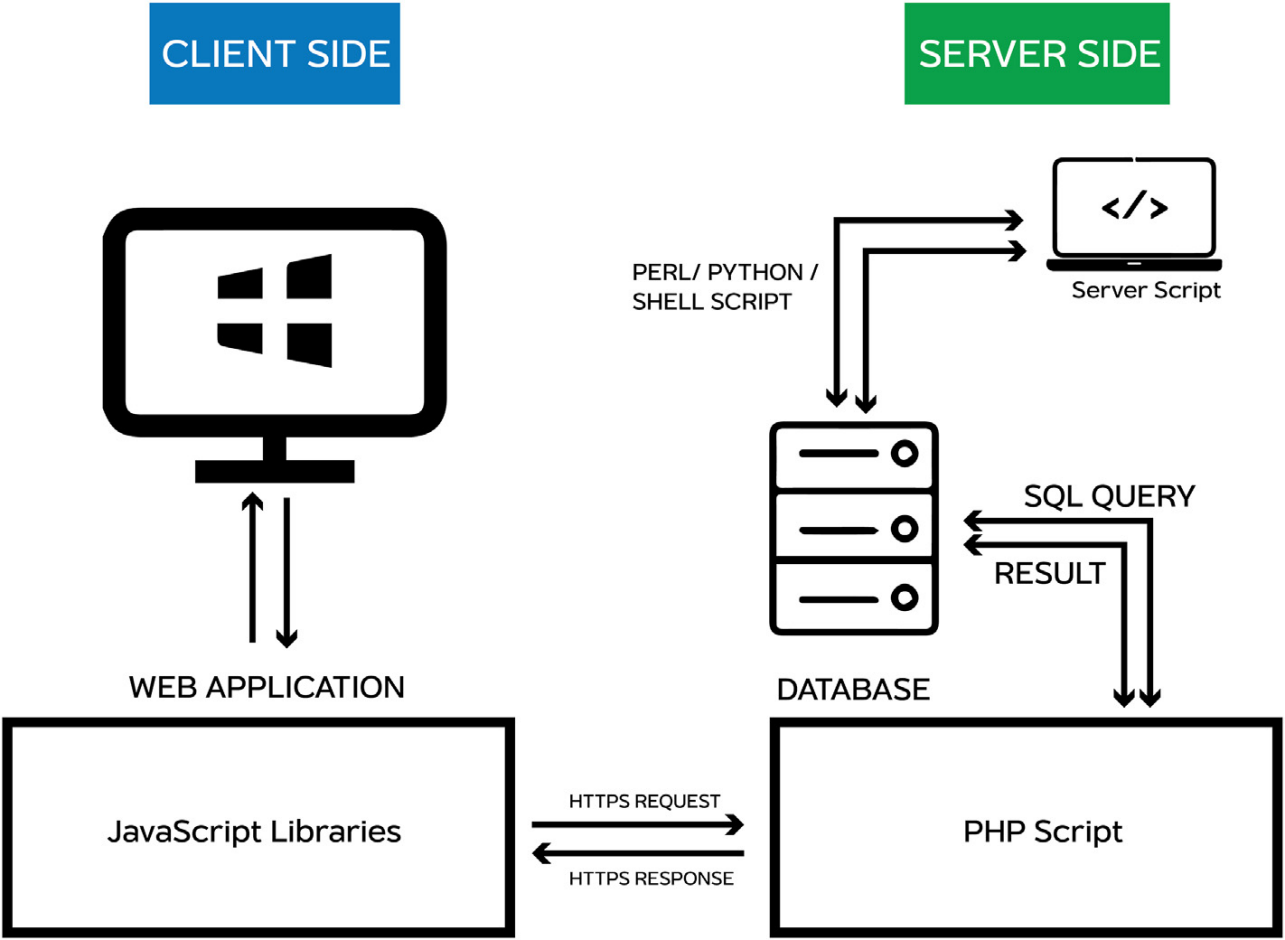
aTAP system architecture.

In addition, the database connection was set up using open-source relational database, MariaDB (https://mariadb.org/), which enables direct client queries from the server. The system architecture of aTAP is visualized in Figure 2.

### 2.4 Pipeline customization

The choice of available default parameters of each piece of software in the bioinformatics pipeline was based on literature reviews and the recommendations of each piece of software. This option has been provided for users who do not work in the bioinformatics field and are not familiar with command-line interfaces. However, experienced users can adjust the parameters using the web interface and select their preferred options before submitting the analysis to aTAP.

### 2.5 Result visualizations

The visualizations of the analyzed results were created using several JavaScript libraries for display on web interfaces including Data-Driven Documents (D3) (https://d3js.org/) and DataTables (https://datatables.net/). D3 was used to create various interactive plots including the volcano, MA, and heatmaps, and DataTables was used for visualizing the results in responsive table form. Additionally, we used the Interactive Cluster Heatmap library (InCHlib) [37] to provide interactive heatmap and dendrogram in the aTAP.

### 2.6 Availability

aTAP is freely available to academic users at https://atap.psu.ac.th/. aTAP allows users to create up to two projects. Each user has a storage capacity of 50 GB per account. To run a bigger project, users can request more space by email to the aTAP admin. The analysis results are kept on the server for 30 days.

## 3 Results and discussion

### 3.1 Web graphical user interface

**Figure 3:**
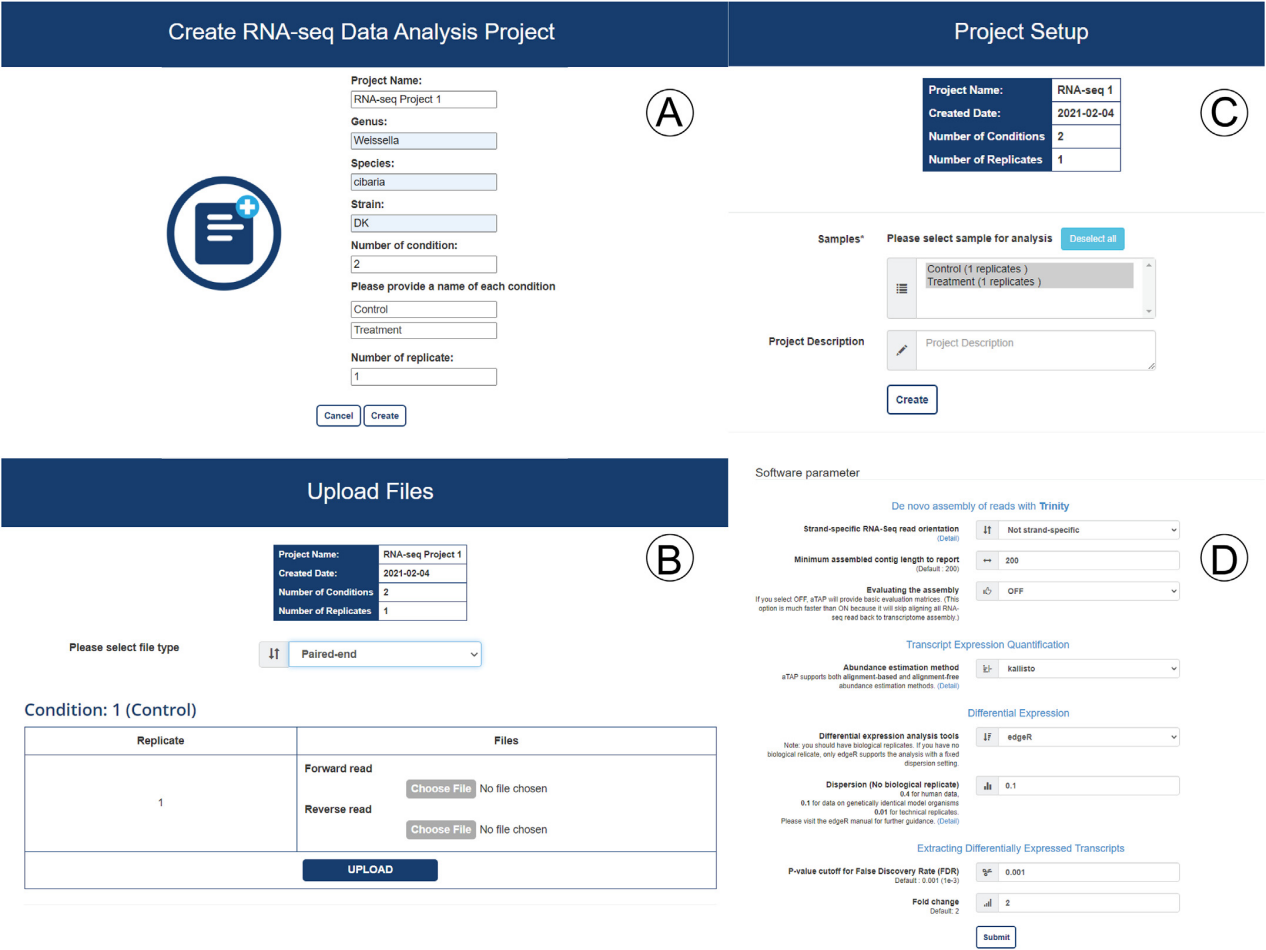
Example pages of aTAP web-based user graphical interface. (A) Create an analysis project; (B) Upload sample files; (C) Select files for performing analysis; (D) Set up the parameters for the analysis.

Users can directly access the aTAP pipeline through an interactive web-based GUI without any need for knowledge of the necessary command lines, as shown in Figure 3. It enables users without high-level computer skills to freely use the bioinformatics pipeline. A personal user account must be created by completing a registration form. Then, the user creates a project by providing basic information about the experiment, including the number of experimental conditions, number of replicates, names of the genera and species of the samples, condition names, etc. This information is used to create metadata for the analysis in each project. Before submitting the analysis, users have to upload the short-read sequencing data to the server via the hypertext transfer protocol secure (HTTPS) protocol in the web interface as shown in Figure 3A aTAP only supports the compressed archive (.gz) format for raw sequence reads (FASTQ format) due to the capacity limitation of the deployed server (Figure 3B). aTAP supports a maximum of 36 samples per job submission for any given experiment. The maximum file size should not exceed 5 GB and must be compressed into GZIP format before uploading to the aTAP server Finally, users have to customize and configure the parameters or leave them at the default values before submitting a request to the aTAP queue (Figures 3C and 3D).

### 3.2 Pipeline customization

Users can customize the bioinformatics pipeline according to their preferences by changing the analysis methods/tools or adjusting several parameters. The parameters for the RNA-seq analysis in aTAP are categorized into (1) *De novo assembly* (2), *Transcript Expression Quantification* (3), *Differential Expression*, and (4) *Extracting Differentially Expressed Transcripts*, as shown in Figure 3D.

In the *De novo assembly* category, the users can set several options, such as “Strand-specific RNA-Seq read orientation”, “Minimum assembled contig length to report”, “k-mer size”, and “Evaluating the assembly”. The descriptions and recommendations are provided in more detail on the linked webpage and help users to select the best parameters for their experiments. In *Transcript Expression Quantification*, users can select an abundance estimation method for the analysis. aTAP supports both alignment-based and alignment-free methods for quantification. Alignment-based tools align all sequences to the transcriptome of a sample. Then, the mapped reads are processed further by computing and counting the number of reads that can map to a transcript or gene [38]. However, this method consumes a lot of computational time and machine resources in terms of main memory and the number of CPU uses. In aTAP, we offer Expectation-Maximization (RSEM) [26] for users who want to use the alignment-based method. On the other hand, alignment-free methods use the k-mer frequencies within the transcriptome libraries to estimate the abundance of a transcript or gene [39]. This method can be performed quickly and consumes less memory and computational time than alignment-based methods. For the alignment-free method, aTAP provides two tools: Kallisto [28] and Salmon [27].

In the *Differential Gene Expression* section, users can select differential expression (DE) analysis tools depending on their samples and whether there are biological replicates in the experiments or not. If there are biological replicates, aTAP provides two methods for calculating the differential expression: DESeq2 [29] and edgeR [30]. If there are no biological replicates in the project, users can only select edgeR. The edgeR dispersion value has to be set before proceeding to further steps. The values for the dispersion parameter must be selected carefully since it directly affects the DE analysis and can cause computational biases [40].

Finally, in the *Extracting Differentially Expressed Transcripts* configuration, users have to provide the P-value cutoff and fold change values to retrieve only the significantly differentially expressed transcripts for plotting and visualizing the results. After the configuration is completed, the workflow can be sent into the processing queue of aTAP. Then, the server-side starts running the submitted job automatically or waits until it reaches the front of the queue for execution. The users can monitor the progress of the project and view the results of each project directly through the web browser.

### 3.3 Analysis results

**Figure 4:**
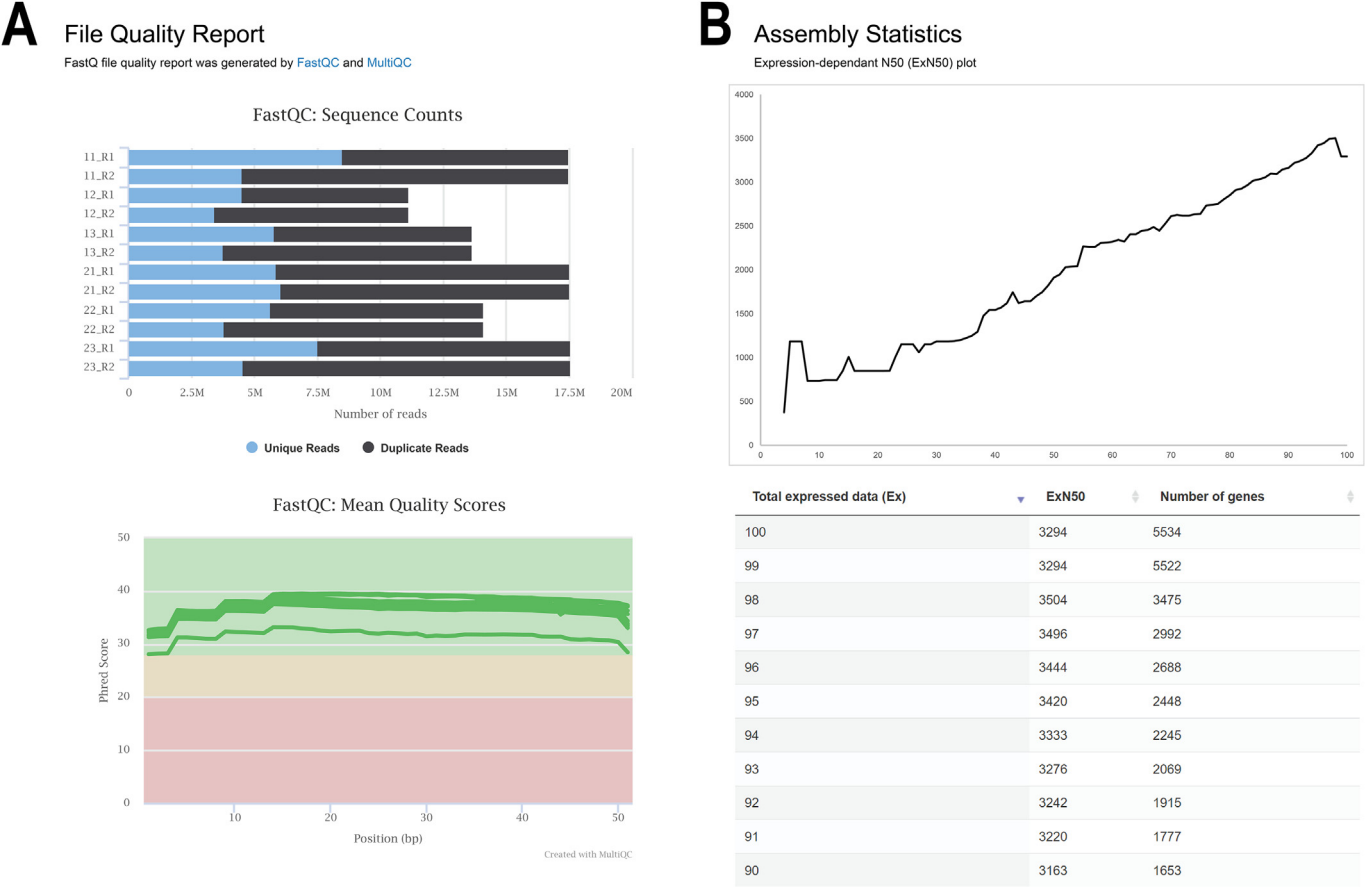
Quality control report and de novo assembly statistics generated by the aTAP system. (A) Quality control report produced by FastQC and multiQC. (B) Assembly statistics based on *de novo* assembly by Trinity.
- **De novo assembly statistics.** The raw sequence reads are loaded into the pipeline to perform a de novo assembly of each sample. aTAP automatically removes low-quality reads before starting the next process. Only the remaining high-quality reads are used to assemble the reads into contigs. When the de novo assembly is complete, the expression-dependent N50 (ExN50) is plotted against a fraction of the total expressed data (Ex) (Figure 4B). These values offer information about the assembly quality in terms of the contiguity and distribution of contig lengths, which can indicate how good the de novo assembly has been.
- **Differential gene expression plots.** In this section, volcano and MA visualizations are plotted using differential expression results calculated by an R script using several coding libraries. The dots in the plots are displayed in different colors to highlight the significantly differentially expressed transcripts/genes in the analysis, as shown in Figure 5A. The visualizations can be directly explored to view transcript/gene information, and high-resolution images can be exported as PNG files.

The analysis results are illustrated in various categories for easy readability and interpretation and can be interactively investigated. Interesting transcripts or related information can be explored directly through the web browser by previewing the information of the displayed transcripts and adjusting the visualization of the heatmap, for example. The analysis results are given as follows:

- **Quality control report.** This report is generated by FastQC and multiQC [21], as shown in Figure 4A. The overview of the sequence quality and general statistics are provided for users in a single tab. Additionally, the results can be explored to view further information such as the sequence length distribution and sequence quality histograms.

**Figure 5:**
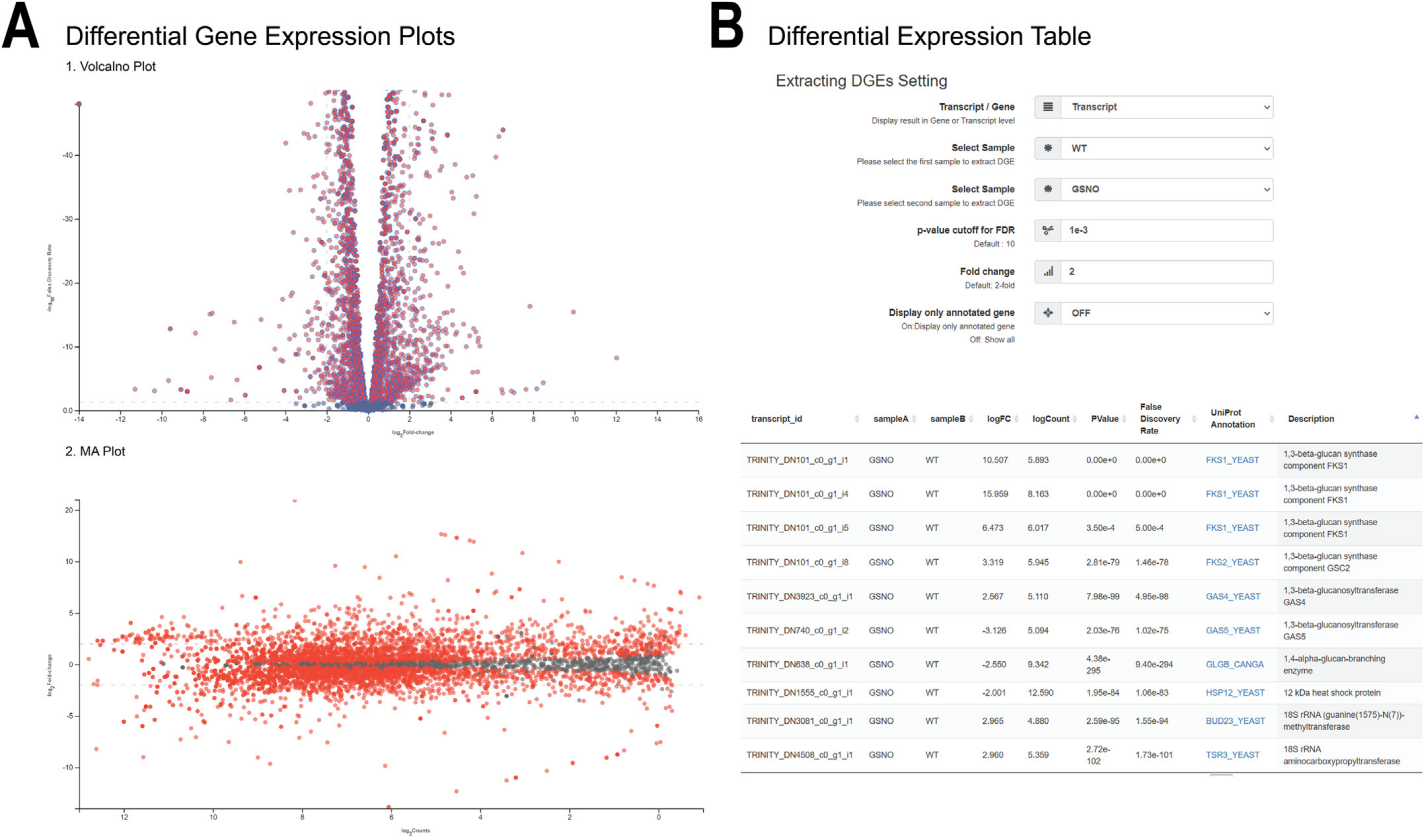
Differential expression results created by the aTAP system. (A) Volcano and MA plots for each of the pairwise comparisons performed. (D) Pairwise differential expression table.
- **Differential gene expression table.** The interactively searchable differential gene expression (DGE) data is visualized in a tabular style, as shown in Figure 5B. The user can explore the information of each transcript, including the log fold change (logFC), log counts per million (logCount), P-value, false discovery rate (FDR), and functional annotations. The aTAP platform also provides a link to additional annotation information in the UniProt database. Users can click to directly view further detail and search target genes using the database search box. The DE table can be easily exported in Excel (.xlsx) and comma-separated value (.csv) format.
- **Heatmap.** The aTAP application automatically extracts differentially expressed transcripts and generates a user-customized heatmap, as shown in Figure 6A. By default, the thresholds are set to at least 4-fold differentially expressed and P-values less than 1E-3. The color and number of the transcripts/genes to display can be freely configured before exporting the image.

**Figure 6:**
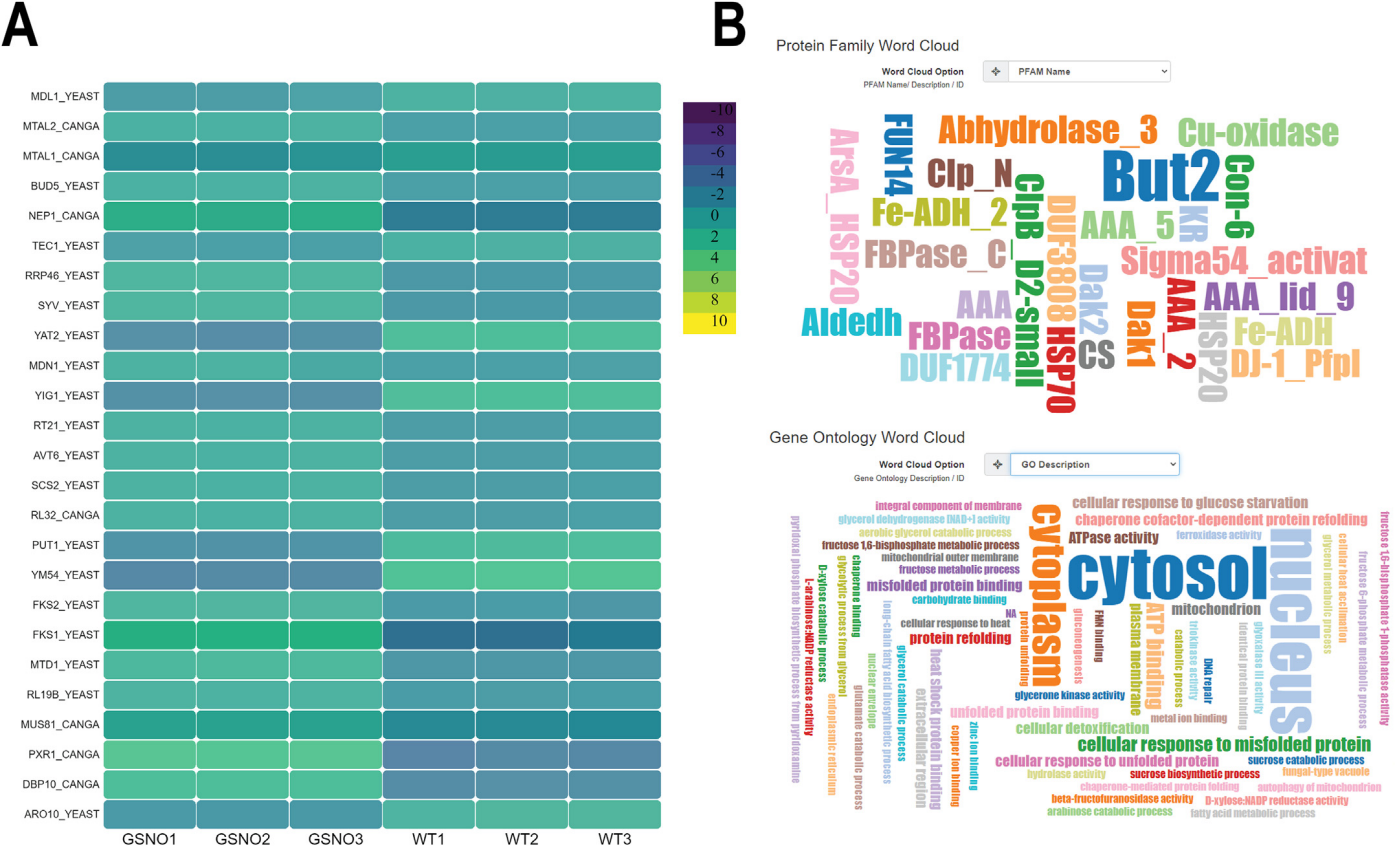
Heat map and functional annotation report. (A) Heatmap of clustering differentially expressed transcripts. (B) Functional annotation of assembled transcripts/genes in word cloud representation and up-/downregulated gene plot.
- **Functional annotation.** The visualizations in the comprehensive report include annotation tables based on protein family (PFAM) and gene ontology (GO) prediction, word clouds, and an up-/downregulated gene bar chart, as shown in Figure 6B. The table can be configured, and interesting annotation names and functional descriptions can be searched for. Moreover, users can access more information directly from the PFAM and GO website via the link given for each annotation. aTAP provides a visual representation from which users can extract the up-/downregulated genes using word clouds.

### 3.4 aTAP performance and comparison of RNA-seq analysis pipelines

aTAP has been validated on several datasets to test the overall performance of the system. To test the analysis of a dataset without replicates, we used the study of Paopradit et al. [41]. They examined the effect of *Vibrio alginolyticus* in quorum sensing-controlled phenotypes of acute hepatopancreatic necrosis disease using microbiological approaches and RNA-seq. Two experimental conditions were analyzed for gene expression, including *Vibrio parahaemolyticus* treated with and without *V. alginolyticus*. The file sizes of four paired-end sequences ranged from 500 to 900 MB (accession numbers SRR12398138 and SRR12459902). With the default parameter settings, aTAP reported that 490 genes were differentially expressed, of which 200 were up-regulated and 290 were down-regulated Analyzing the same data with uTAP [12], MAP-RSeq [13] and aRNApipe [14] generated 524, 540, and 538 differentially expressed genes, respectively. These three tools were tested with default parameters, using *V. parahaemolyticus* RIMD 2210633 (NC_004603 and NC_004605) as a reference. aTAP generated the lowest number of expressed genes since it used the de novo method to assemble the transcripts and may misassemble and/or produce meaningless contigs but the expressed genes generated by aTAP overlapped 95%, 96% and 95% with uTAP, MAP-RSeq and aRNApipe, respectively.

For a dataset with replicates, we used a study on the RNA-Seq-based analysis of the transcriptomic landscape of *Candida glabrata* [42]. This study provided the details of a gene expression analysis of *C. glabrata* in nutrient-rich media (WT) and under nitrosative stress (GSNO). Twelve paired-end Illumina read files of the two conditions, with three biological replicates for each (accession numbers SRR1582646-SRR1582648, SRR1582649-SRR1582651) were loaded into aTAP to perform de novo assembly and functional annotation. The application took ∼4.5 h to process ∼12 GB of data. Furthermore, we also ran aTAP with the *Solanum lycopersicum* dataset [43], consisting of 12 samples from four experimental conditions. This study presented a transcriptome analysis of tomato fruits under heat stress at 42 °C for 0 h, 24 h, 48 h, and 96 h, respectively. Each condition had three replicates and file sizes ranged from 1.7 to 2.5 GB (in gzip archive). The assembly, quantification, DE analysis, visualization and reporting were completed in ∼16.5 h. From these two jobs, the overlapping of expressed genes with expressed genes generated by uTAP, MAP-RSeq and aRNApipe was around 90–92%, 92–95%, and 94–96%, respectively. Meanwhile, the total number of differentially expressed genes generated by aTAP was slightly smaller than the numbers generated by the other tools, by around 1.5–2.7%.

Specifications of bioinformatics pipelines for RNA-seq analysis were compared and the findings are presented in Table 1. Generally, the other platforms are specially designed for normalization and differential expression analysis [15, 16, 17]. They do not support the assembly or alignment process, which still requires text-based commands to install and run the pipelines. Moreover, some tools require installation using CLI [12, 14]. This could be a significant problem for novice users when setting up the tool and learning how to use it precisely. Only the uTAP and aTAP offer both GUI and comprehensive bioinformatics analysis starting from the assembly to the DE analysis process in a single tool. However, the uTAP still requires installation with CLI using the docker platform, which is very difficult for non-bioinformaticians to complete step by step. In this study, we proposed a platform for analyzing RNA-seq data from the assembly step to the visualization step. Users without computer skills can complete their tasks using only the GUI. Also, aTAP offers predefined parameters to help users configure their projects in a few easy steps.

**Table 1:** Comparison of RNA-seq analysis pipelines.

Tool	GUI	Installation with CLI	Prerequisite software	Hosting	Comprehensive analysis∗	Interactive report	Reference
uTAP	Yes	Required	Yes	Local	Yes	Yes	[12]
MAP-RSeq	No	Required	Yes	Local	Yes	Yes	[13]
aRNApipe	No	Required	Yes	Local	Yes	Static report	[14]
TCC-GUI	Yes	No	No	Remote	No (only DE)	Yes	[15]
GENAVi	Yes	No	No	Remote/Local	No (only DE)	Yes	[16]
RNASeqGUI	Yes	Required	Yes	Local	No (only DE)	Static report	[17]
aTAP	Yes	No	No	Remote	Yes	Yes	This study

∗ Comprehensive analysis: Comprehensive bioinformatics analysis includes data quality control, trimming, assembly/mapping, differential gene expression analysis, and preparation of reports.

## 4 Conclusions

aTAP is an integrated platform combining various bioinformatics tools and pipelines into a single web application that enables research communities to analyze their transcriptome data without needing to recruit bioinformatics and computer science skills. aTAP provides a completely automated workflow for comprehensive bioinformatics analysis, starting from quality control to de novo transcriptome assembly and differential expression calculation. Furthermore, aTAP allows users to interact with the processed information and plots, which helps researchers to easily explore and discover new findings from their experiments.

## Declarations

### Author contribution statement

Komwit Surachat: Conceived and designed the experiments; Performed the experiments; Analyzed and interpreted the data; Contributed reagents, materials, analysis tools or data; Wrote the paper. Todd Duane Taylor: Conceived and designed the experiments; Wrote the paper. Wanicbut Wattanamatiphot; Sukgamon Sukpisit; Kongpop Jeenkeawpiam: Performed the experiments.

### Funding statement

Dr. Komwit Surachat was supported by Thailand Research Fund [Grant No. MRG6280229] and the National Science, Research and Innovation Fund (NSRF) and Prince of Songkla University (Grant No. SCI6505013S).

### Data availability statement

Data included in article/supp. material/referenced in article.

### Declaration of interest’s statement

The authors declare no conflict of interest.

### Additional information

No additional information is available for this paper.
